# Organic Juice Processing Quality from the Processors’ Perspective: A Qualitative Study

**DOI:** 10.3390/foods12020377

**Published:** 2023-01-13

**Authors:** Lisa Marie Borghoff, Carola Strassner, Christian Herzig

**Affiliations:** 1 Faculty of Agricultural Sciences, Nutritional Sciences and Environmental Management, Justus Liebig University Giessen, 35390 Giessen, Germany; 2 Department of Food · Nutrition · Facilities, FH Münster University of Applied Sciences, 48149 Münster, Germany

**Keywords:** organic food processing, organic food quality, juice processing, expert interviews, Germany, Austria, ProOrg

## Abstract

Organic food quality is based on processing. While the EU organic production regulation focuses on agricultural production, private standards provide more detailed information about further processing. For the development of organic processing, practitioner perspectives can provide valuable input. To get insight into practitioner perspectives, we conducted semi-structured expert interviews with nine employees of seven partly organic juice processing companies from Germany and Austria. Interview topics were (i) quality of organic juice processing in general, (ii) assessment of specific processing techniques, (iii) product quality of organic juice and (iv) flow of information between producer and consumer. We conducted a thematic analysis. We found that the experts’ understanding of process quality mostly includes more aspects than the EU organic production regulation. It covers the whole food chain plus aspects of social and environmental sustainability. The experts prefer directly bottled juice of local raw materials but chiefly accept juice made from concentrate of exotic raw materials because of environmental concerns. Organic juice is preferred when it is cloudy and natural fluctuations are interpreted as an indicator of natural quality. The experts report that consumer information is challenging because of low food literacy. Raising this might help reduce the number of processed juices on the market.

## 1. Introduction

Organic food production follows an integral product identity paradigm [1]; it focuses less on specific product attributes and more on the manufacturing process [2,3,4]. Organic food quality, therefore, includes aspects of the product and the production process [5]. Moreover, organic food production should guarantee health, ecology, fairness and care, as described in the basic principles of IFOAM [6]. Nielsen [7] elaborated this approach with the duty of care towards the product, the people and the environment. According to Kahl et al. [5], the processing of organic raw material into consumer food products [8] should be in line with these principles and preserve the valuable components of the organic raw material [9] p. 21, [10]. However, our understanding of organic food quality is still limited, and more elaborated definitions of processing methods are needed [11]. Although organic food production in the European Union is regulated by EU Regulation 2018/848, it also contains only limited information on specific processing technologies and even the regulations of the organic farming associations only partly provide more detailed guidance on specific processing technologies.

In practice, processors are limited by procedural, legal and economic restrictions when choosing a food processing technology [12] and they point to challenges in finding suitable processing technologies for organic food [11]. Processors have steadily developed their own perspective on food quality [13], so their expert knowledge can be utilised for the further development of organic food production. Still, studies on processors are scarce, compared to other food chain actors [14]. Among the very few examples are studies by Kretzschmar and Schmid [11], Seidel and Kretzschmar [12] and Górska–Warsewicz et al. [15] on organic food, and by Ilbery and Kneafsey [13] on food quality and processing that include processors in their study participants. Thus, in our paper, we give insight into the quality perception of organic food processors by presenting the results of semi-structured expert interviews we conducted with employees of partly organic juice processing companies from Germany and Austria in 2020. In doing so, this article contributes to the field of organic juice production and food quality perceptions.

The paper provides, firstly, an introduction to the juice market in Germany and Austria, whereby more market data is available for Germany than for Austria. Then we move on to organic juice processing and related processing standards and issues. After this, materials and research methods are explained. The presentation of findings is structured according to processing quality and assessment, product quality, information flow between producer and consumer, and further aspects including raw materials, guidelines and food trade. The findings are finally discussed before a conclusion is given.

## 2. Background

Juices are generally consumed in significant quantities in Germany and Austria. While there is a decline in Germany in both the per capita consumption of fruit juice (from 42 L in 2002 to 30 L in 2020) [16] and the number of fruit juice producers (from 455 in 2000 to 311 in 2021) [17], the consumption of vegetable juice has increased from 0.97 L per capita in 2002 to 1.9 L in 2020 [18]. Demand for fair trade and organic juice is increasing in Germany [19,20]. In Austria, the per capita consumption of fruit juice was 21.4 L in 2021 [21]. The largest turnover of single-variety juice in Germany and Austria is achieved with orange juice (€2.68 billion and €189.10 million, respectively, in 2022). In second place in both countries is apple juice (€1.87 billion in Germany and €84.18 million in Austria). Grape juice, pineapple juice and grapefruit juice are also popular single-variety juices. Other high turnover is achieved through juice mixtures, smoothies and juices from other raw materials such as vegetables (Germany: 3.76 billion €; Austria: 125 million €) [17,22]. In Germany, orange juice is mainly sold as juice from juice concentrate, while for apple juice the ratio between direct juice and juice from juice concentrate is about equal (as of 2017) [19].

Juice is a source of micronutrients (e.g., vitamins) and bioactive phytochemicals (e.g., carotenoids, flavonoids) [23,24,25]. As a plant-based product, it can be beneficial for health when consumed in moderate amounts [26,27] or processed further, for example, into probiotic food [25]. The juice composition depends on the type of raw material (e.g., cultivar) and the processing method [28,29]. The cultivation system (organic or non-organic) also influences the raw material properties but the cultivar and environment have a stronger effect [30]. In general, organic fruits and vegetables have a higher amount of dry matter and phenolic compounds, and less nitrate, heavy metals and pesticide residues compared to non-organic products [31,32]. The optimal maturity level of the raw material is one of the key factors for the quality of the final juice [33]. To preserve the valuable components of the juice, processing should be as mild as possible but also guarantee food safety.

Juice processing in general includes cleaning, the extraction of the juice (e.g., via shredding and pressing) and a preservation process. Juice can either be bottled directly (not from concentrate, NFC juice) or undergo a process of concentration and reconstitution (from concentrate, FC juice) [34]. Juice concentrate offers benefits with regard to shelf life and transportation efficiency [35]; however, the process affects the food properties [36]. Juice normally has turbidity, including dietary fibre such as pectin [37]. Turbidity can either be stabilized, leading to cloudy juice, or removed, leading to clear juice [38]. Juice processing includes thermal stress and oxygen contact. Both negatively affect the juice quality [24,36,39]. Thermal stress can partly be replaced by pressure, e.g., with high-pressure pasteurization (HPP) [39,40]. The negative impact of oxygen contact can be reduced by oxygen exclusion, inactivation of browning enzymes or the addition of browning inhibitors, such as ascorbic acid [41].

Food processing is always a complex process, as the characteristics of raw materials are subject to natural fluctuations, e.g., depending on the variety or season [42,43]. In the case of some fruit varieties, fluctuations occur naturally (biennial or alternate bearing) [44]. For processors, this means that they must always adapt the process to the material [43]. Fluctuations are particularly challenging for processors of organic products: Demand for organic raw material is growing, leading to increased competition [15]. Harvests are also at risk from weather conditions [15] and harvest volumes of organic products are subject to greater fluctuations due to restrictions in the handling of fertilisers and pest control [45]. Organic juice producers can compensate for variances in the quality of the raw material by blending, but they need corresponding quantities of raw material to do so. Compensation through additives, on the other hand, is only possible to a limited extent [12].

Organic juice producers are faced with the challenge of producing a product that meets the requirements of the law and the consumer, while at the same time retaining the special organic quality. For this organic quality, some processing aspects are particularly critical. Leskinen and Särkkä-Tirkkonen [46] identified key issues regarding the principles of naturalness and authenticity, environmental sustainability and social aspects, and appropriate technology for several food products. Table 1 gives an overview for the case of juice production with a selection of additional points for the principle of environmental sustainability.

Some, but not all of these issues are reflected in the different guidelines for organic juice processing. In the EU Council Regulation 2018/848 and in the regulations of the Austrian organic farming association Bio Austria, there are no specific regulations for the processing of fruit or vegetable juices [53,54]. The regulations of other organic farming associations are partly more specific and restrictive (see Table 2).

The production of FC juice is prohibited by all tabulated organic farming associations, except for Naturland which may give permission when it makes sense based on the ecological balance sheet [60] (pp. 2, 3). The production of juice made from concentrate is more efficient with regard to transportation, which is an important contributor to the ecological footprint of juice [61,62]. In the case of transportation efficiency, there might be a conflict between the principle of naturalness and authenticity with the principle of environmental sustainability. The same applies to the use of enzymes that can raise yields but are questionable regarding naturalness [63,64].

To understand how processors deal with these challenges and to analyse their understanding of quality, an explorative study was conducted, which is presented next.

## 3. Materials and Methods

Due to the low number of studies on this topic, we decided to conduct a qualitative, exploratory study based on semi-structured expert interviews with employees of at least partly organic juice processing companies [65]. We developed the interview guideline in a team and conducted two pre-tests in partly organic food processing companies [66]. We included the topics (i) quality of organic juice processing in general, (ii) assessment of specific processing techniques for organic juice, (iii) product quality of organic juice and (iv) flow of information between processor and consumer in the interview guideline. The interview guideline followed a structure but was open to changes and spontaneous or company-specific questions within the interview [67]. We included questions about the experts’ understanding of careful processing because this term is used in the EU-organic production regulation and asked the experts for a guiding principle for organic juice processing. At the end of each interview, we asked the experts if they wanted to add any remaining important aspects in their point of view [66]. A translated version of the interview guideline is available as supplementary material. The translation was prepared by the first author.

We planned to conduct interviews with employees from different departments of at least four organic or partly organic juice processing companies in German-speaking countries. As a further criterion, we set a limit of at least 50 employees per company to avoid having departments managed by the same person. This limit was set after consultation with the Association of Organic Food Processors (AÖL). After contacting several potential companies at Biofach 2020 in Nuremberg the COVID-19 pandemic arose in Europe and we had to change our method from face-to-face to telephone interviews. Moreover, many juice processing companies were affected by reduced work schedules, and we could only conduct interviews with a maximum of 1–2 employees per company. We decided to include more than four companies in our research and remove the limit of 50 employees per company to raise the amount of data points. We were able to conduct interviews in six fruit companies and one vegetable juice processing company before we ended the data collection in December 2020.

We interviewed eight employees from quality and safety, product and production management, and product and quality development positions (employees E1, E2 and E4–E9) and one interview with an employee from the field of distribution (employee E3). The interviews were conducted by the first author and ranged from 28 min to 1 h and 37 min, with a median length of 42 min. They were transcribed and directly pseudonymised by the first author and by a professional service, with transcription rules according to Dresing and Pehl [68]. The interviews were conducted, transcribed, and analysed in German; all quotations within this article are translations by the authors. The interview partners did not get a donation for their participation, and all agreed in writing to the recording and processing of their data.

The companies the interview partners work in can be classified as micro, small and medium-sized enterprises (SMEs) according to their number of employees [69]. The juice processing sector is very heterogeneous, but on the whole it is characterised by SMEs [70]. Therefore, the selection of companies is in line with the sector. However, due to the qualitative design, no representativeness is possible. All companies hold the EC organic certificate, but no certificate from an organic farming association. All companies produce organic and non-organic products. The companies produce a wide range of different pure juices and juice mixtures. They produce NFC und FC in cloudy and clear quality as well as juice nectar. They are located in Germany and Austria.

The analysis of the interviews followed the qualitative text analysis approach by Kuckartz [71] and was performed with MaxQDA 2020. We used a set of concept-driven main and sub-codes based on the interview guideline to start the analysis. During the coding process, we developed data-driven main and sub-codes based on the interview transcripts. Data-driven main codes are the quality of the raw material, claims of the trade, and guidelines for processing. Data-driven sub-codes refer to specific aspects of processing, such as thermal stress or oxygen contact. Food miles and packaging are further data-driven sub-codes associated with environmental sustainability. We developed the code system in teamwork to raise its quality [72].

## 4. Results

Table 3 gives an overview of the main results of the interviews according to the interview topics. The following subchapters offer more detailed information of the results.

### 4.1. Quality of Organic Juice Processing in General

The experts describe the processing of organic and non-organic juice as similar because the relevant guidelines (fruit juice regulation, Association of the Industry of Juices and Nectars (AIJN) guideline) do not differ between organic and non-organic products:

“It doesn’t matter whether you process organic fruit juice or fruit juice. The fruit juice regulation applies to both.”

(E1, p. 4)

They describe the protection of organic authenticity by avoidance of contamination and ensuring traceability as the most important aspects. This makes up “most of the work that comes to a juice processing company with organic goods” (E5, p. 14). In their parallel processing companies, some experts do this with one “organic day” per week. They also report the stricter limitation of ascorbic acid addition for organic juice as a further difference.

The experts describe the process quality as depending on the quality of the raw material and the protection against product damage. They say that processing should be fast and value preserving, with few processing steps of low intensity, e.g., low thermal stress, enzyme-free pressing or production of cloudy NFC juice instead of clarified FC juice. Some experts name this “natural processing”:

“As little processing as possible, so directly pressed, for example, no filtering of the juice. As little intervention as possible, mechanically pressed without the use of enzymes and things like that, yes? No use of chemicals. That’s what I mean by honest, natural.”

(E7, p. 11)

“Directly, gently, quickly, perhaps. In other words, as few steps as possible, as little treatment as possible, as close to nature as possible.”

(E6, p. 20)

Some experts add less transportation, local and environmentally friendly production as well as working safety and fairness in their perception of high processing quality. They use the following keywords to describe a guideline for processing: natural, safe (food safety and working safety), honest, clean and tidy, ecologically sustainable, local and fair.

Some experts criticize the terms gentle and careful processing as marketing phrases or redundant because carefulness is always required in fruit processing (organic as well as non-organic), also for economic reasons:

“I don’t think there is a food producer who doesn’t process their food—even conventional food—with care. […] Because they would reduce the quality, they would reduce the shelf life, they would reduce the possible sales price.”

(E1, p. 3)

In general, the experts define careful processing as production in line with all the relevant guidelines, a strict separation of juice types and a clean, hygienic and safe production that leads to a high-quality of the final product.

The experts describe gentle processing as gentle to the product, with which they mean preserving sensory value and colour, and protection against decay. They achieve this with a reduction of thermal stress and oxidation and short transportation and storage times. They describe choosing the correct temperature as an act of balance:

“[…] on the one hand the end product should be processed as little as possible […]. On the other hand, we cannot avoid pasteurization to preserve the product […]. So, it’s a bit of a balancing act.”

(E7, p. 4)

One expert describes the balance between necessary heat and product damage as “quantum satis” (E4, p. 5–6). The experts achieve the balance with modern machinery and automatic steering and control, e.g., heat-efficient plate pasteurizers. However, one expert adds that local, handmade production in a small company can also be gentle, even if the thermal stress is higher due to old-fashioned production technology. This would be a different type of gentleness:

“[…] there are still a few small cideries […] that produce their own apple juice and then perhaps apply greater heat. But I would still speak of a very gentle treatment because I would simply argue that I have my own apples, that they don’t have to be transported over a hundred kilometres in boxes and stored for several days. I can wash them myself by hand and don’t need machines and so on. And I could also justify this with regionality, for example.”

(E6, p. 18)

Besides single processing steps, the experts stress that the whole production chain, from the contact to the farmers until the final product, is important for quality.

During the interview with expert E1, the topic of natural variances of the raw material emerged and was described as critical for processing. We included this topic in the interview guideline, given that knowledge of ingredient variability is necessary for operations planning (cf. van der Spiegel et al. [42], Riddick et al. [43]).

Regarding all interviews, the experts describe how natural variance of the raw material emerges, e.g., due to weather conditions during growth and that variances are challenging because they aim to produce homogenous products, as exemplified by the following statement of an employee:

“It doesn’t help consumers if the juice is sour today and the next pack they buy is sweet or mild or something. If there are fluctuations somewhere, that’s always bad.”

(E6, p. 22)

The experts report that it is possible to harmonise variances of juice with blending (mixing of several juices from different harvest periods), but this requires double pasteurization: first before storing the juice in bulk and a second time after bottling. This thermal stress hurts product quality. The experts report that one could also work with homogenous raw materials from long-term partnerships to reduce variances, which would be easier for smaller companies because of the smaller quantity required. They note that it would also be possible to use mixed raw materials, e.g., from orchard meadows (scattered fruit trees of high nature conservation value [73]), that harmonise variations. The experts reject interventions for standardization because they are not in line with organic quality and “[…] an organic product is also allowed to show the variability of nature.” (E5, p. 7)

The experts raise the topic of local production and food miles during the interviews. Some experts prefer raw materials from local production to support their local area and avoid raw materials from countries they are critical of for production methods. Moreover, they see local products as a strategy in the juice market. For the experts, as few food miles as possible are important for the quality of organic products, both in terms of the raw materials and the finished juice. They describe tensions in this area regarding products with long transportation distances because they are not grown locally. One expert discusses the difference between local and organic products and their environmental benefits:

“Is conventional from the region better than organic from Egypt, for example? […] Local or organic? […] Just because it’s organic doesn’t necessarily mean it is environmentally friendly.”

(E7, p. 20)

### 4.2. Assessment of Specific Processing Techniques for Organic Juice Processing

All experts stress the importance of the reduction of thermal stress and oxygen contact. Ascorbic acid is used as a browning inhibitor, but some experts assess high ascorbic acid amounts as unnatural. They propose to reduce the necessary amount of ascorbic acid by low oxygen contact during production because “that goes a bit against naturalness. And of course, you don’t want all juices to be brown. […] But maybe some people should consider whether they can get by with half the vitamin C” (E6, p. 18). They use closed systems and protective gas during milling, pressing and storage to decrease oxygen contact. For milling, the experts report that pneumatic presses lead to less oxygen contact than band presses but are not as efficient. One expert prefers extraction with a decanter for an even larger reduction of oxygen contact.

To reduce thermal stress, the experts prefer direct bottling because this requires only one pasteurisation step. However, they add that directly bottled juice cannot be blended and so natural variances cannot be harmonised with different charges. The experts report that high-pressure pasteurization leads to lower thermal stress, but this juice is less stable regarding colour. They refer to the active cooling of bottled juice as another way to reduce thermal stress.

When asked about any legal processing technologies that are not in line with their organic quality understanding, only some experts refer to technological examples. They see blending fresh juice with older ones and brightening juice with active carbon or high doses of ascorbic acid as critical. Some experts assess post-extraction (pomace is juiced a second time under the influence of enzymes) critically but one expert notes that it leads to fewer food losses, which is preferable for organic food.

Most experts report that NFC juice has a better sensory impression but is more expensive than FC juice, which limits its usage. The experts describe NFC juice as more honest than FC juice and easier to produce in organic quality because of the simple process:

“The sensory quality is better, and the production process is much more natural […]. It’s simply a more honest, more direct product, […].”

(E7, p. 5)

“[…] the easiest way to have a one hundred per cent authentic organic product is direct juice, because if you have some kind of organic nectars […] that makes the production much more complicated and much too prone to error.”

(E8, p. 3–4)

Consumers would also prefer organic juice as NFC. Besides these positive aspects, the experts criticised NFC juice made from exotic fruits and discussed if it is more energy efficient to work with FC juice in these cases. Some experts accept FC juice for organic food while others do not find it in line with organic, even if it is less sustainable in some cases. The experts propose to check the carbon footprint and produce NFC juice preferably from local raw materials. They also report that one can raise the quality of FC juice by diluting the concentrate with less water than required. One expert states that “today’s concentrate plants are so well made, including the aroma recovery, that even professional sensory experts can’t tell if it’s direct juice.” (E1, p. 13)

The experts associate organic juice with cloudiness. They see clearing as an unnecessary processing step and an old-fashioned beauty standard. They describe the cloudy juice as “more natural, in colour, in taste, […]” (E1, p. 6). Further problems of clearing are the thermal stress of the process and gelatine as a non-vegan clearing additive. But not all experts see clearing as a problem and do accept it for organic juice:

“I see no reason why organic should automatically be cloudy.”

(E6, p. 20)

The experts raised lactic-acid fermentation as a further processing technology, especially for vegetable juice. As advantages of this process, they describe the more natural taste compared to blending with lemon juice, less thermal stress, easier digestion, simplicity, and cost efficiency. One expert adds that fermentation in general leads to products of high quality and “all top-class things are ultimately made in this form of fermentation” (E8, p. 8). As disadvantages, they report hygienic challenges, time intensity and a higher number of processing steps.

A further topic brought into the interviews by the experts is the packaging material for juice. The experts describe the sustainability of the packaging as an important topic within the companies but also from the consumers’ point of view. They discuss the environmental aspect of the packaging within the companies or with B2B customers:

“Packaging is always a point of discussion. […] This is a question that we deal with very often: Is it compatible with organic quality?”

(E7, p. 10)

In addition to being environmentally friendly, the packaging should protect the juice and be easy for the consumer to handle.

### 4.3. Product Quality of Organic Juice

The experts describe taste and colour as key quality parameters which should be as close to the raw material as possible. A juice should “still smell[s] and taste[s] like a fresh carrot, for example. And it should also have the same colour as far as possible” (E4, p. 9,10). Taste and colour are sensitive to thermal stress and oxidation, so these should be as low as possible during processing. The experts also assess quality by the sugar and acid content of the raw material, which is influenced by the growing conditions and fluctuates from harvest to harvest.

The experts describe the main difference between organic and non-organic juice on the level of product characteristics as the content of pesticide residues:

“With organic juices, the main issue is the absence of pesticides. In terms of taste and ingredients, there is no reason why they should be better than conventional products. Their colours are just as beautiful as the conventional products.”

(E4, p. 11)

Only two experts address issues of the different taste of organic juice: one expert describes organic vegetable juice as herbier; the second expert states that the different sensory impressions would come from the knowledge of drinking an organic product.

### 4.4. Flow of Information between Producer and Consumer

The experts report that the expected consumer requirements influence the way of processing, e.g., producing clear or cloudy organic juice. One expert reports that they do not use enzymes made with genetic engineering even for their non-organic products because consumers strongly reject genetically modified organisms (GMOs). However, the experts also report on improving communication with the consumer: They try to explain processing to the consumer, e.g., why there are natural variations. Some customers consider them as a sign of reduced quality, while others accept variances, especially if they want a natural product:

“Especially loyal customers who buy the product regularly will notice. And some understand that there are simply natural fluctuations, and others […] don’t understand. They think we’ve done something to the quality […]”

(E7, p. 8)

The experts report a generally low food literacy from consumers; this influences how they talk about processing, e.g., it would be better to talk about vitamin C than ascorbic acid. The experts describe challenges in adequately presenting information about the product and process quality; too much information might be overwhelming, and it is better to concentrate on key characteristics. The experts see a potential to use company websites for more neutral and transparent information, e.g., presenting a live stream of the production process.

The experts report that only a few consumers ask questions about processing techniques and more on the nutritional value, allergens and gluten or alcoholic content, additives, the origin of the raw material, the packaging material and if the juice and/or packaging is vegan. Some experts report irritation about the nature of consumer enquiries, e.g., the question about gluten content. However, they do not blame the consumer, because of the complexity of food technology. One expert describes guided tours as an efficient tool to raise interest and knowledge of food processing. The experts would like the consumers to know about the difference between FC juice, NFC juice and nectar; that organic does not mean 100% free from pesticide residues due to ubiquitous contaminations, the region of origin of the raw material and the history of the company that processes the juice.

### 4.5. Further Aspects

#### 4.5.1. Raw Material

The experts report the high relevance of the raw material for the quality of the final juice and one expert refers to the raw material in their guideline for good food processing:

“Always make sure that the raw materials you use are flawless, that they are certified organic.”

(E4, p. 9)

They describe the purchase of only certain cultivars as one strategy to achieve high quality. They find the purchase of high-quality raw material challenging, especially for large companies that need high amounts of fruit and vegetables. They expect this to become even more challenging in the future due to climate change:

“We really have to start thinking about climate change, about increasing desertification and droughts. We are already noticing all this.”

(E6, p. 10)

#### 4.5.2. Guidelines

The experts describe the German fruit juice regulation (FrSaftErfrischGetrTeeV) as a base for both organic and non-organic juices and they find further details in the AIJN guidelines. Some of the experts report that this guideline already leads to natural and careful processing.

The experts assess the EU organic production regulation differently. Negative effects of the regulation are the higher workload due to traceability and the ban of pea protein for clearing making animal-based gelatine necessary:

“That’s why it’s such a pity that pea protein still hasn’t made it. […] it was just bad luck at the time that pea protein came out shortly after the organic regulation.”

(E5, p. 13, 14)

One expert considers the EU organic production regulation to be incomplete; its gaps would be filled by the organic farming associations. Besides these negative aspects of the EU organic production regulation, the experts positively mention that the strictness of the regulation makes organic juice processing quite simple, and one expert proposes even to further limit the permitted non-organic ingredients in processed organic foods. This case is regulated by article 30 (5); a maximum of 5% of the ingredients in processed organic food may come from conventional production, and only if these ingredients are listed in the respective Annex of the regulation.

Some experts prefer working with their own quality standards that are “always over and above the guidelines” (E9, p. 21). Practical examples are the reduction of reuse and using less water than required for the dilution of fruit juice concentrate.

#### 4.5.3. Requirements of the Food Trade

The experts report that the food traders can set the prices and that these support the tendency to make the juice producers exchangeable: food traders would prefer every juice producer to make the same products. The food traders do not support lactic-acid fermented juices and instead, they demand the production of cheap nectars. Regarding production standards, some food traders require the IFS Food certificate from processors.

## 5. Discussion

### 5.1. Quality of Organic Juice Processing in General

In line with [10], the experts report that processing should be value preserving, e.g., with low temperature. They describe organic processing with aspects we can find in the principle of naturalness and authenticity and include also environmental aspects such as food miles [46]. They also include aspects of working safety and fairness in their perception. Based on the IFOAM principles and further guidelines for organic food production, Nielsen [7] describes this approach as carefulness towards the product, the environment and the people. Carefulness towards the people is important especially for juices made from raw materials from the so-called Global South (see [74]) because of the often precarious working conditions in the growing countries [75]. To the best of our knowledge, research on the social dimension of organic juice processing is scarce. We found only two more studies on this topic: One study performed in a citrus-producing area in Spain found no benefit in social sustainability after conversion to organic [76] while another study performed in Kenya found certified organic farms to have better support and training for their workers [77]. Research on the social hot spots of food production can help identify relevant problems. For example, Du et al. [78] identified health and safety, labour rights and decent work as the most critical aspects for the case of sugar cane production in Brazil. We propose to extend the research, e.g., using Social Life Cycle Assessment (S-LCA) tools, to find out more about the social sustainability of organic juices [79,80].

The experts’ definition of gentle processing is mostly in line with the definition of careful processing by the experts from Kretzschmar and Schmid [11], who meant “the maximum to keep the important compounds and […] to avoid undesired compounds or nutritional losses” (ibid, p. 113). Only one expert added that handmade, local processing can be seen as its own type of gentleness.

Like Gallmann [10] proposes, the experts’ quality perception included not only single processes but the whole production chain. Carefulness starts with the growth of the raw material, so there is a need for good contact with the farmers. The experts consider this as an aspect of sustainability and a way to face the challenges of climate change and natural variances.

Variations occur naturally with juice [44] and the possibilities for food standardisation are more sharply limited in the organic sector [12]. In addition, the greater fluctuations in harvest volumes for organic products [45] can limit opportunities to compensate for variations. Most of our experts proposed to accept natural variances because they belong to a natural product and every form of standardisation (blending, producing juice from concentrate) leads to product damage. Instead, the experts propose to communicate the naturalness of variances to consumers. Based on our interviews and the results from Seidel and Kretzschmar [12], we consider adding the handling of natural variances as a further key issue for organic fruit and vegetable processing regarding the principle of naturalness and authenticity (see Table 1) [46]. More research is needed about the stabilization of harvests in organic farming, e.g., with better pest control, optimal timing of fertilisation and adapted plant breeding [45,81].

Food miles were an important topic for process quality from the experts’ perspective, in line with Leskinen and Särkkä-Tirkkonen [46]. They assess short food miles and local production as ways to support the local area and environmentally friendly production. The problem with local cultivation is that it is only possible in an environmentally friendly way with varieties that are suitable for the location. Species that are not suited to the location require the use of energy-intensive techniques such as greenhouses [2]. Therefore, exotic raw materials sometimes have to be transported from far away. In this case, some of our experts see reducing food miles as a legitimate reason to use FC juice, just like the organic farming association Naturland [60] p. 6, while for others, the greater change brought about by the concentration process is seen as incompatible with organic quality.

### 5.2. Assessment of Specific Processing Techniques for Organic Juice Processing

Regarding specific processing technologies, the experts named problematic areas similar to Leskinen and Särkkä-Tirkkonen [46]. However, they were open to discussing techniques that are associated with greater environmental friendliness, such as using enzymes to increase juice production and reduce waste or FC juice. The possible benefits of FC juice regarding food miles are presented above, and the experts also report FC juice being less expensive than NFC juice. This can be explained by more efficient transportation and packaging for FC juice [82]. But the experts also describe the production of FC juice as more complicated and error-prone than the simple process of NFC juice. Fruit juices are among the most frequently adulterated foods and are hard to protect due to natural variances of the raw material [83], so the desire for procedures that are less prone to deviations in product quality is understandable. Besides the question of using or not using FC juice, most of the experts preferred cloudy organic juices but did not reject clear juices in general. The question of whether to clarify organic juice or not is also still unclear at the level of the organic farming associations (see above) and remains an area to discuss for organic food. Cloudy juice has higher amounts of beneficial ingredients [37], so it would meet the consumer expectation of organic foods to be healthier [84]. Also, cloudy juice can be interpreted as more natural because it contains more of the original ingredients of the raw material and has been produced with fewer processing steps [85].

The experts raised and discussed the topic of packaging in the context of environmental protection. They reported that this topic is also important for consumers. The organic farming associations already address this issue, with some having detailed guidelines for the packaging of certain products [55] (pp. 103–108), [56] p. 5, [58] p. 12, [59] p. 9, [86] (pp. 119–122, 125), [87] p. 25, [88] p. 8, [89] p. 13, [90] p. 30. In contrast, the EU organic production regulation addresses packaging only in terms of food safety [53] p. 31. A stronger regulation of packaging within the EU organic production regulation could ensure greater uniformity and would do justice to the high importance of this issue for processors and consumers. On the other hand, the current regulation gives processors leeway to use the packaging they consider most appropriate. In doing so, they can rely on studies that analyse and compare the environmental impact of different packaging (e.g., [48]).

### 5.3. Product Quality of Organic Juice

The optimal flavour of juice depends on the ripening grade of the raw material [33]. The optimum degree of ripeness is determined by the sugar–acid ratio which the experts describe as a key indicator of product quality that they use in practice. Since raw produce is perishable, it is important to process it quickly. Plant protection products can reduce post-harvest losses, but their type and quantity is strictly limited in organic farming [81,91]. Sufficient quantities of raw materials of the desired quality must be available at the right time for processing. The experts describe climate change as a challenge for this. The literature describes both possible positive and negative consequences of climate change on the cultivation of fruit and vegetables (e.g., changes in taste and appearance) [92]. Changes in the composition of raw materials also affect the taste of juice. Manufacturers could respond to this with greater standardisation. However, as natural products are desired, the changes are perhaps inevitable and remain so. As in the case of passing on natural variations to the juice, an attempt could be made to increase consumer acceptance of climate change-induced effects on organic food quality by providing information accordingly.

### 5.4. Flow of Information between Producer and Consumer

The experts reported challenges in communication with the consumer because consumers would be less interested in technology than in the nutritional quality of the juice or suitability to a certain diet. Hüppe and Zander [85] also found a lack of knowledge about food technology in their focus groups. Consumers are affected by framing in their perception of food processing [93]. Pictures of idyllic production might be misleading, as one of the experts points out, and it is better to use neutral information. This might increase trust in food production and the organic food market [94,95] and fulfil the need for transparency [96] which is especially high for consumers that are sensitive towards sustainability [97]. Information on processing should be carried out with consumer-adequate language [98] (p. 117), [98] (pp. 41–50), as the interviewed experts already do in the case of ascorbic acid.

Some of the experts would like the consumers to know that FC juice is more sustainable when coming from exotic raw materials. This might be challenging because, in the German market, NFC juice is more often labelled with claims of sustainability than FC juice, as Klink et al. found in a market study from 2014 [99]. In general, they found only relatively few juices with information on environmental (e.g., organic label) or social sustainability (e.g., fair trade label), so consumers may not be aware of these topics. However, this could have changed in the meantime due to the increased demand for organic and fair-trade juice. A current market survey could provide new insights here.

### 5.5. Further Aspects

The experts’ understanding of the processing quality of organic juice includes not only the EU organic production regulation but also aspects of the guidelines from the organic farming associations (e.g., preference for cloudy juice, environmentally friendly packaging), even if none of the SMEs held a certificate from an organic farming association at the time of the interviews. While they follow all relevant guidelines, they also practice their own quality standard higher than the basic regulations. This was also reported by the experts questioned by Seidel and Kretzschmar [12]. Practitioners are relevant experts for the future development of organic food processing and therefore should be included in future research.

The experts report how the power of the food trade influences their work. Processors must respond to the demands of the trade [2]. Those power imbalances can lead to unfair trading practices (UTP) [100] (pp. 22–23), [101] p. 14. These can be prevented by long-lived and personal relationships [101] (pp. 10, 15). Further research into the cooperation between processors and traders seems to be useful to investigate the topic of UTP in more detail.

The interviews show that the interviewed processors associate organic processing with care for the product, people and the environment. There are tensions between these aspects of care in organic juice production, especially between care for the product and care for the environment. In practice, a trade-off takes place in the choice of technology. The experts understand care as relevant throughout the food chain. The assumed demand of consumers and the power of the trade influence the type of processing. The difficulties in communicating with consumers mentioned by the experts could be countered by more neutral information in an appropriate language.

## 6. Conclusions

In this study, we investigated the understanding of processing quality from the perspective of processors. Our findings show that the experts include aspects of organic food processing that go beyond the basic regulations laid down in the EU organic production regulation in their quality perception. The experts stress the importance of environmental sustainability for organic juice. Some describe a conflict between care for the product and care for the environment, especially in the case of juice made from concentrate. A discussion between different stakeholders from the organic sector about the weighting of individual aspects of organic processing quality could contribute to solutions here. The standards of the growers’ associations already provide detailed information on processing in some cases, and the standard-setters can thus make a valuable contribution to the development of the field of organic processing quality. The EU organic production regulation could undergo further development in this respect and take up further aspects that have not been included so far but are already advocated by the practitioners in our study.

Further research is needed to find processing methods that are careful of the product, the environment and the people. Evaluation methods also need to be developed to assess the extent to which methods are achieving these goals. Care for the product is achieved primarily through modern technology. Supply chains, supplier relationships and occupational safety in the company are particularly important to achieve care for the environment and for people.

The experts in this study prefer low-processed juices that reflect the natural variations of the raw material. They describe the placement of these products as challenging, in part because consumers expect standardised products and interpret fluctuations as a lack of quality. Increasing consumer knowledge about juice production could help to increase the acceptance of less processed products. Educational institutions could develop offers for this purpose.

Overall, our study shows that processors are relevant partners for the further development of organic processing. They have a clear and differentiated view of organic processing and know the challenges of translating the standards into practice. We therefore suggest the inclusion of processors in further research in this area in order not to neglect this valuable perspective, and that they also be engaged in the context of regulatory development processes.

## Figures and Tables

**Table 1:** Overview of key issues from the field of juice processing with relevance to the organic principles.

Organic Principles	Naturalness and Authenticity	Environmental Sustainability	Social Dimension and Appropriate Technology
Key Issues	Reconstitution of fruit juices	Conditions of raw material cultivation (e.g., use of fertilizers and pesticides, production in greenhouses, etc.) Food miles (longer transport distances) Waste reduction Packaging	Traditional processing technologies
	Filter and clarification techniques		Regionally adopted small processing plants and technologies
	Use of additives and processing aids		
	Use of antioxidants		
	Use of ion exchange treatment		
	Use of enzymes		

Own, shortened table based on [46] (pp. 56–57), [2,47,48,49,50,51,52].

**Table 2:** Overview of organic standards for juice processing.

Processing Technology	Council Regulation (EU) 2018/848	Organic Standards According to German Organic Associations					
		Demeter	Bioland	Biokreis	Bio-park	Gäa	Naturland
FC-juice	(+)	(−)	(−)	(−)	(−)	(−)	(*)
Clarification	(+)	Preference for cloudy juices	(*)	(+)	(+)	(*)	(+)
Addition of ascorbic acid	(+)	(−)	(*)	(*)	(*)	(*)	(*)

(+) = permitted; (−) = not permitted; (*) = Permissible under certain conditions or for certain products; Own table based on: [55] (pp. 108–111), [56] (pp. 4–5), [57] (pp. 3, 5), [58] (pp. 28–29), [59] p. 38, [60] (pp. 2, 3).

**Table 3:** Overview on the main results for each interview topic.

Interview Topic	Main Results
Quality of organic juice processing in general	Protection of organic authenticity is most important Quality includes the whole production chain as well as aspects of environmental and social sustainability Processing should be fast and include few processing steps of low intensity Modern technology and automation ensure product protective processing Natural variances in the raw material are challenging and require more intense processing
Assessment of specific processing techniques	Preference for the reduction of oxygen contact instead of ascorbic acid addition in high doses to inhibit browning Direct bottling of NFC juice needs less thermal stress but leads to variable product characteristics Blending compensates natural fluctuations but requires more thermal stress FC juice requires more thermal stress, but offers ecological advantages with exotic raw materials Clearing is predominantly rated as inappropriate for organic juice
Product quality of organic juice	Colour and taste are the key quality parameters and should be as close to the raw material as possible Main difference between organic and non-organic juice is the content of pesticide residues
Flow of information between processor and consumer	Consumer ratings influence juice processing (e.g., clearing of juice) Low consumer food literacy challenges information sharing
Raw material	Raw material is crucial for juice quality Procurement is affected by climate change
Guidelines	The existing guidelines provide impetus for natural processing, but can still be expanded in some areas Companies use own standards
Requirements of the food trade	Retailer demand influences juice processing and quality standards

## Data Availability

The data presented in this study are available on request from the corresponding author. The data are not publicly available due to privacy reasons.

## Supplementary Materials

The following supporting information can be downloaded at: https://www.mdpi.com/article/10.3390/foods12020377/s1.
