# Supplementary material for: Organic Juice Processing Quality from the Processors’ Perspective: A Qualitative Study
Source: Foods. 2023 Jan 13;12(2):377. doi: 10.3390/foods12020377 (PMC9857823; doi:10.3390/foods12020377)
Supplement: Supplementary file 1 [file foods-12-00377-s001.zip › foods-2118107-supplementary.pdf]

Basic Interview Guideline

This document shows the basic interview guideline. The order of the questions was set after the pretest but could be flexibly adapted to the course of the interview if necessary [67]. When interviewees used unclear descriptions of processing, such as the term "natural", they were always asked to explain what they understood by these terms [66].

Topic 1: Process Quality

Background: In this part of the interview, we want to find out about the experts' understanding of process quality, especially regarding organic juice. This includes their interpretation of the term careful processing which is used in the EU organic regulation (EC) No 2018/848, their perception of the general direction of processing and limitations of processing – especially for organic juice.

Questions:

- What is your understanding of the term careful processing?
- How is care implemented in the production of juice?
- How do you choose which processing methods to use for your juice?
- Can you give a guiding principle of what high-quality processing should look like?

Topic 2: Specific processing techniques

Background: There are different techniques for the production processing of juice (e.g., clarification, production of juice made from concentrate). In this part of the interview, we want to find out about the experts' views on these processing techniques, especially with regard to the question of whether they fit in with organic quality. In this part of the guideline we added company-specific questions about the processing technologies in use [67].

Questions:

- Which processing technologies are (not) suitable for organic processing from your perspective?
- How do you deal with natural fluctuations?

Topic 3: Product Quality

Background: Processing affects the product characteristics. Therefore, conclusions about the necessary processing can be drawn from the desired product properties. We want to find out about the experts' understanding of product quality, especially for organic products.

Questions:

- What are the key characteristics for high juice quality?

- Are there characteristics that, in your view, do not suit an organic juice?

Topic 4: Flow of information between producer and consumer

Background: In this part of the interview, we want to find out more about the communication between producer and consumer.

Questions:

- When you get enquiries from customers, what do they want to know about your organic juice?
- What do your customers want to know about the processing steps?
- How do you decide which information you use to advertise it, e.g., on the packaging or the homepage?
- What do you think your customers should know about your juice and its production in general?

Finale:

Background: The interview ends with an open question about missing important aspects [66].

Question:

- Are there any other aspects of processing organic juice that are important to you but you haven't told me yet?

66 Galletta, A. Mastering the Semi‐Structured Interview and Beyond: From Research Design to Analysis and Publication; New York University Press: New York, NY, USA, 2013; ISBN 9780814732953.
67 Adams, W.C. Conducting Semi-Structured Interviews. In Handbook of Practical Program Evaluation, 4th ed.; Newcomer, K.E., Hatry, H.P., Wholey, J.S., Eds.; Jossey-Bass: San Francisco, CA, USA, 2015; pp 492–505, ISBN 9781119171386.
